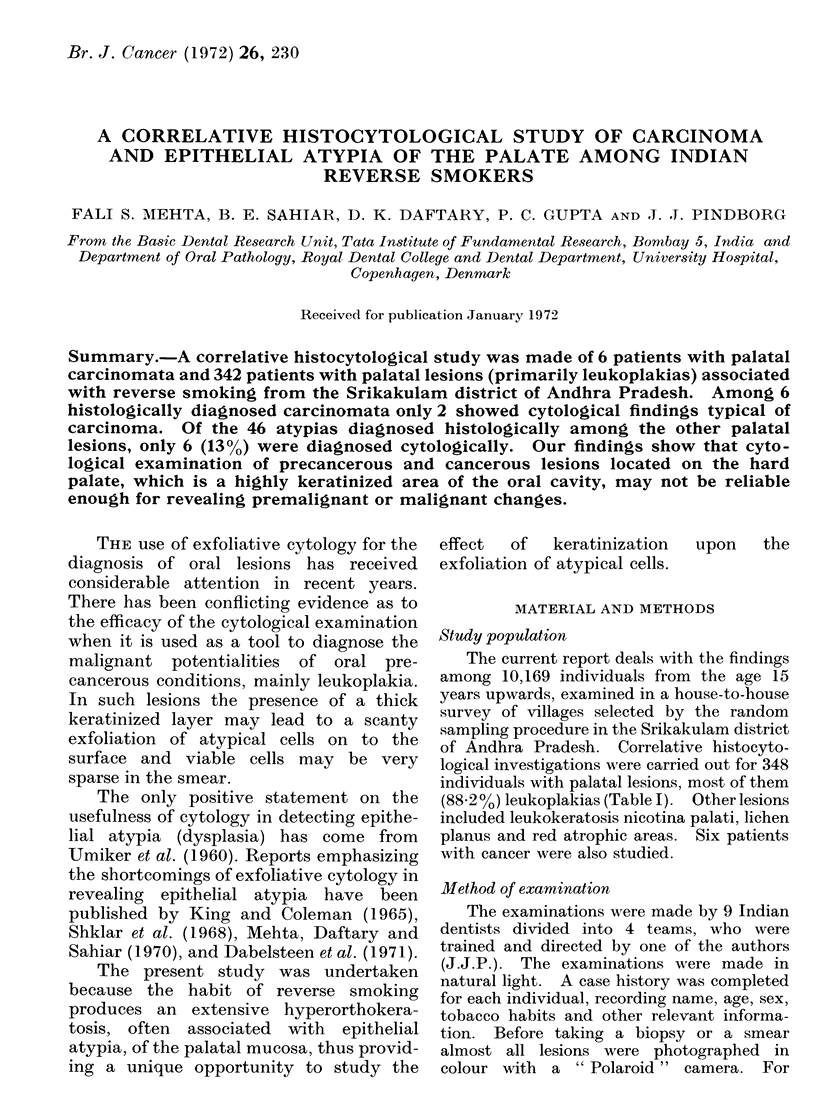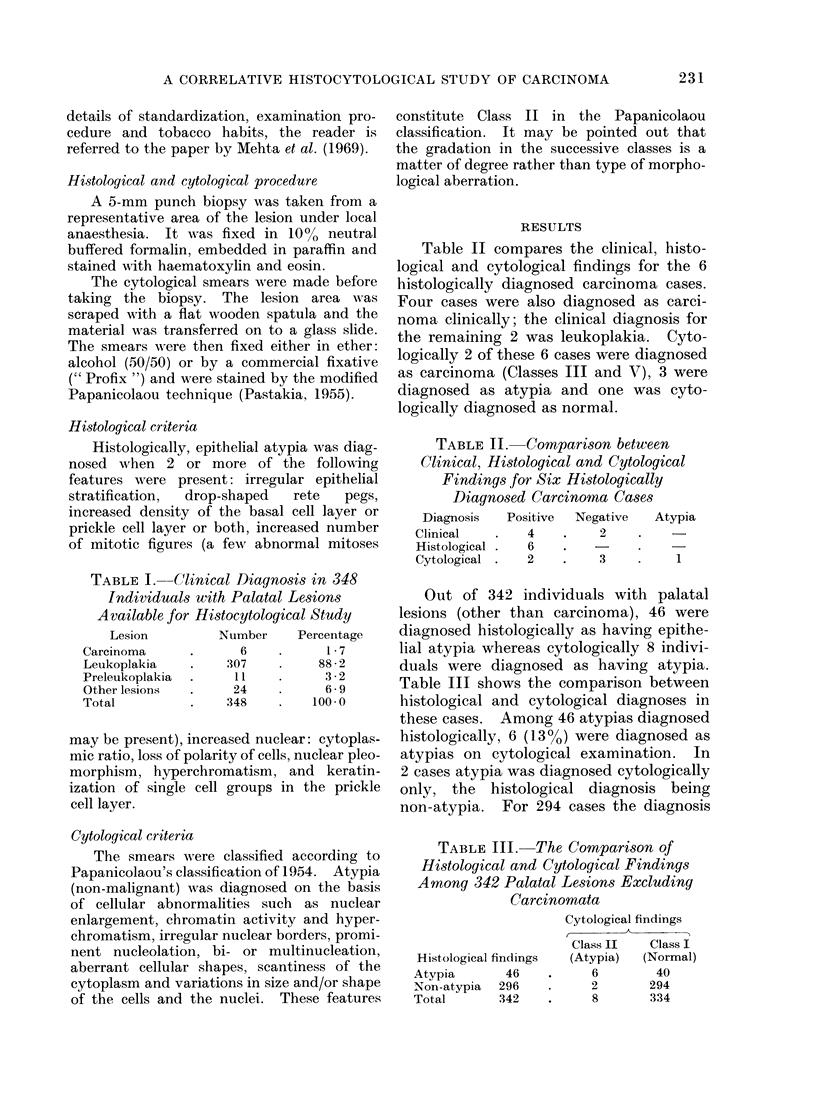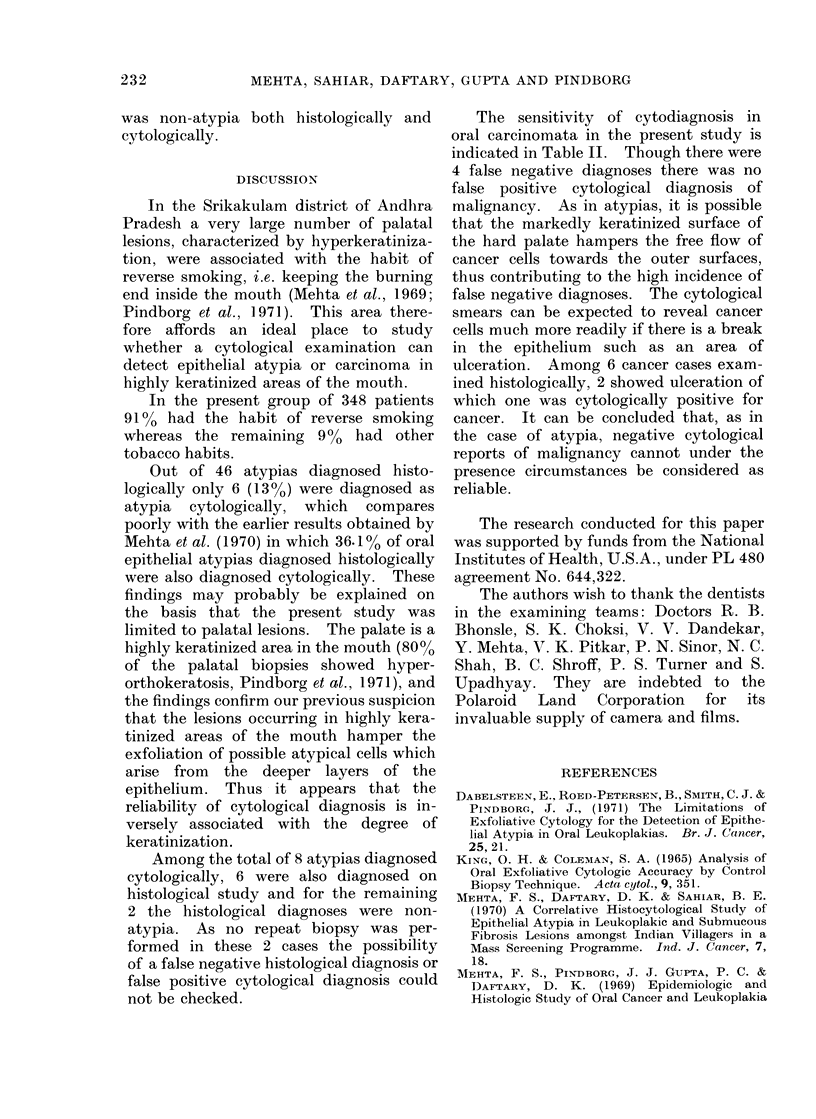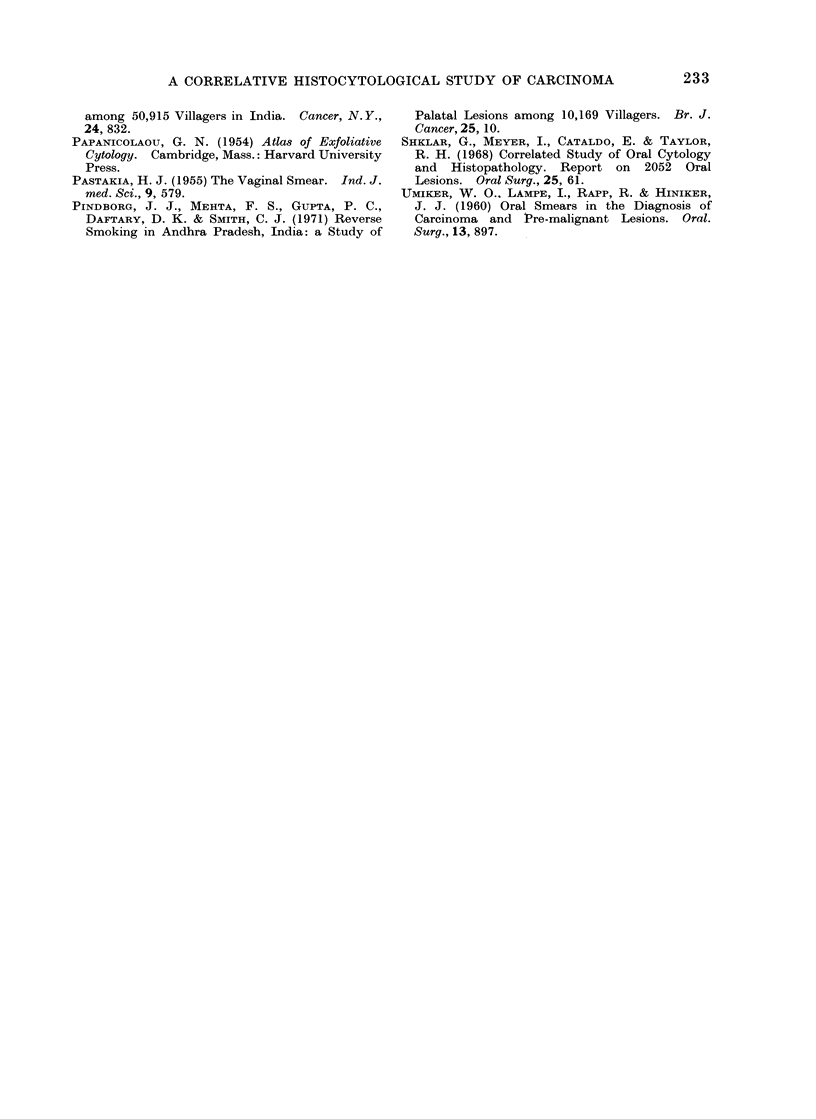# A Correlative Histocytological Study of Carcinoma and Epithelial Atypia of the Palate Among Indian Reverse Smokers

**DOI:** 10.1038/bjc.1972.31

**Published:** 1972-06

**Authors:** Fali S. Mehta, B. E. Sahiar, D. K. Daftary, P. C. Gupta, J. J. Pindborg

## Abstract

A correlative histocytological study was made of 6 patients with palatal carcinomata and 342 patients with palatal lesions (primarily leukoplakias) associated with reverse smoking from the Srikakulam district of Andhra Pradesh. Among 6 histologically diagnosed carcinomata only 2 showed cytological findings typical of carcinoma. Of the 46 atypias diagnosed histologically among the other palatal lesions, only 6 (13%) were diagnosed cytologically. Our findings show that cytological examination of precancerous and cancerous lesions located on the hard palate, which is a highly keratinized area of the oral cavity, may not be reliable enough for revealing premalignant or malignant changes.


*Br. J. Cancer* (1972) **26**, 230

### A CORRELATIVE HISTOCYTOLOGICAL STUDY OF CARCINOMA AND EPITHELIAL ATYPIA OF THE PALATE AMONG INDIAN REVERSE SMOKERS

FALI S. MEHTA, B. E. SAHIAR, D. K. DAFTARY, P. C. GUPTA AND J. J. PINDBORG

*From the Basic Dental Research Unit, Tata Institute of Fundamental Research, Bombay 5, India and Department of Oral Pathology, Royal Dental College and Dental Department, University Hospital, Copenhagen, Denmark*

Received for publication January 1972

**Summary.—A correlative histocytological study was made of 6 patients with palatal carcinomata and 342 patients with palatal lesions (primarily leukoplakias) associated with reverse smoking from the Srikakulam district of Andhra Pradesh. Among 6 histologically diagnosed carcinomata only 2 showed cytological findings typical of carcinoma. Of the 46 atypias diagnosed histologically among the other palatal lesions, only 6 (13%) were diagnosed cytologically. Our findings show that cytological examination of precancerous and cancerous lesions located on the hard palate, which is a highly keratinized area of the oral cavity, may not be reliable enough for revealing premalignant or malignant changes.**

THE use of exfoliative cytology for the diagnosis of oral lesions has received considerable attention in recent years. There has been conflicting evidence as to the efficacy of the cytological examination when it is used as a tool to diagnose the malignant potentialities of oral precancerous conditions, mainly leukoplakia. In such lesions the presence of a thick keratinized layer may lead to a scanty exfoliation of atypical cells on to the surface and viable cells may be very sparse in the smear.

The only positive statement on the usefulness of cytology in detecting epithelial atypia (dysplasia) has come from Umiker *et al.* (1960). Reports emphasizing the shortcomings of exfoliative cytology in revealing epithelial atypia have been published by King and Coleman (1965), Shklar *et al.* (1968), Mehta, Daftary and Sahiar (1970), and Dabelsteen *et al.* (1971).

The present study was undertaken because the habit of reverse smoking produces an extensive hyperorthokeratosis, often associated with epithelial atypia, of the palatal mucosa, thus providing a unique opportunity to study the effect of keratinization upon the exfoliation of atypical cells.

### MATERIAL AND METHODS

### *Study population*

The current report deals with the findings among 10,169 individuals from the age 15 years upwards, examined in a house-to-house survey of villages selected by the random sampling procedure in the Srikakulam district of Andhra Pradesh. Correlative histocytological investigations were carried out for 348 individuals with palatal lesions, most of them (88·2%) leukoplakias (Table I). Other lesions included leukokeratosis nicotina palati, lichen planus and red atrophic areas. Six patients with cancer were also studied.

### *Method of examination*

The examinations were made by 9 Indian dentists divided into 4 teams, who were trained and directed by one of the authors (J.J.P.). The examinations were made in natural light. A case history was completed for each individual, recording name, age, sex, tobacco habits and other relevant information. Before taking a biopsy or a smear almost all lesions were photographed in colour with a "Polaroid" camera. For details of standardization, examination procedure and tobacco habits, the reader is referred to the paper by Mehta *et al.* (1969).

*Histological and cytological procedure*

A 5-mm punch biopsy was taken from a representative area of the lesion under local anaesthesia. It was fixed in 10% neutral buffered formalin, embedded in paraffin and stained with haematoxylin and eosin.

The cytological smears were made before taking the biopsy. The lesion area was scraped with a flat wooden spatula and the material was transferred on to a glass slide. The smears were then fixed either in ether: alcohol (50/50) or by a commercial fixative (" Profix ") and were stained by the modified Papanicolaou technique (Pastakia, 1955).

*Histological criteria*

Histologically, epithelial atypia was diagnosed when 2 or more of the following features were present: irregular epithelial stratification, drop-shaped rete pegs, increased density of the basal cell layer or prickle cell layer or both, increased number of mitotic figures (a few abnormal mitoses may be present), increased nuclear: cytoplasmic ratio, loss of polarity of cells, nuclear pleomorphism, hyperchromatism, and keratinization of single cell groups in the prickle cell layer.

TABLE I.—*Clinical Diagnosis in 348 Individuals with Palatal Lesions Available for Histocytological Study*

| Lesion | Number | Percentage |
|---|---|---|
| Carcinoma | 6 | 1·7 |
| Leukoplakia | 307 | 88·2 |
| Preleukoplakia | 11 | 3·2 |
| Other lesions | 24 | 6·9 |
| Total | 348 | 100·0 |

*Cytological criteria*

The smears were classified according to Papanicolaou's classification of 1954. Atypia (non-malignant) was diagnosed on the basis of cellular abnormalities such as nuclear enlargement, chromatin activity and hyperchromatism, irregular nuclear borders, prominent nucleolation, bi- or multinucleation, aberrant cellular shapes, scantiness of the cytoplasm and variations in size and/or shape of the cells and the nuclei. These features constitute Class II in the Papanicolaou classification. It may be pointed out that the gradation in the successive classes is a matter of degree rather than type of morphological aberration.

### RESULTS

Table II compares the clinical, histological and cytological findings for the 6 histologically diagnosed carcinoma cases. Four cases were also diagnosed as carcinoma clinically; the clinical diagnosis for the remaining 2 was leukoplakia. Cytologically 2 of these 6 cases were diagnosed as carcinoma (Classes III and V), 3 were diagnosed as atypia and one was cytologically diagnosed as normal.

TABLE II.—*Comparison between Clinical, Histological and Cytological Findings for Six Histologically Diagnosed Carcinoma Cases*

| Diagnosis | Positive | Negative | Atypia |
|---|---|---|---|
| Clinical | 4 | 2 | — |
| Histological | 6 | — | — |
| Cytological | 2 | 3 | 1 |

Out of 342 individuals with palatal lesions (other than carcinoma), 46 were diagnosed histologically as having epithelial atypia whereas cytologically 8 individuals were diagnosed as having atypia. Table III shows the comparison between histological and cytological diagnoses in these cases. Among 46 atypias diagnosed histologically, 6 (13%) were diagnosed as atypias on cytological examination. In 2 cases atypia was diagnosed cytologically only, the histological diagnosis being non-atypia. For 294 cases the diagnosis

TABLE III.—*The Comparison of Histological and Cytological Findings Among 342 Palatal Lesions Excluding Carcinomata*

| Histological findings | | Cytological findings: Class II (Atypia) | Class I (Normal) |
|---|---|---|---|
| Atypia | 46 | 6 | 40 |
| Non-atypia | 296 | 2 | 294 |
| Total | 342 | 8 | 334 |

was non-atypia both histologically and cytologically.

### DISCUSSION

In the Srikakulam district of Andhra Pradesh a very large number of palatal lesions, characterized by hyperkeratinization, were associated with the habit of reverse smoking, *i.e.* keeping the burning end inside the mouth (Mehta *et al.*, 1969; Pindborg *et al.*, 1971). This area therefore affords an ideal place to study whether a cytological examination can detect epithelial atypia or carcinoma in highly keratinized areas of the mouth.

In the present group of 348 patients 91% had the habit of reverse smoking whereas the remaining 9% had other tobacco habits.

Out of 46 atypias diagnosed histologically only 6 (13%) were diagnosed as atypia cytologically, which compares poorly with the earlier results obtained by Mehta *et al.* (1970) in which 36·1% of oral epithelial atypias diagnosed histologically were also diagnosed cytologically. These findings may probably be explained on the basis that the present study was limited to palatal lesions. The palate is a highly keratinized area in the mouth (80% of the palatal biopsies showed hyperorthokeratosis, Pindborg *et al.*, 1971), and the findings confirm our previous suspicion that the lesions occurring in highly keratinized areas of the mouth hamper the exfoliation of possible atypical cells which arise from the deeper layers of the epithelium. Thus it appears that the reliability of cytological diagnosis is inversely associated with the degree of keratinization.

Among the total of 8 atypias diagnosed cytologically, 6 were also diagnosed on histological study and for the remaining 2 the histological diagnoses were non-atypia. As no repeat biopsy was performed in these 2 cases the possibility of a false negative histological diagnosis or false positive cytological diagnosis could not be checked.

The sensitivity of cytodiagnosis in oral carcinomata in the present study is indicated in Table II. Though there were 4 false negative diagnoses there was no false positive cytological diagnosis of malignancy. As in atypias, it is possible that the markedly keratinized surface of the hard palate hampers the free flow of cancer cells towards the outer surfaces, thus contributing to the high incidence of false negative diagnoses. The cytological smears can be expected to reveal cancer cells much more readily if there is a break in the epithelium such as an area of ulceration. Among 6 cancer cases examined histologically, 2 showed ulceration of which one was cytologically positive for cancer. It can be concluded that, as in the case of atypia, negative cytological reports of malignancy cannot under the presence circumstances be considered as reliable.

The research conducted for this paper was supported by funds from the National Institutes of Health, U.S.A., under PL 480 agreement No. 644,322.

The authors wish to thank the dentists in the examining teams: Doctors R. B. Bhonsle, S. K. Choksi, V. V. Dandekar, Y. Mehta, V. K. Pitkar, P. N. Sinor, N. C. Shah, B. C. Shroff, P. S. Turner and S. Upadhyay. They are indebted to the Polaroid Land Corporation for its invaluable supply of camera and films.

### REFERENCES

Dabelsteen, E., Roed-Petersen, B., Smith, C. J. & Pindborg, J. J., (1971) The Limitations of Exfoliative Cytology for the Detection of Epithelial Atypia in Oral Leukoplakias. *Br. J. Cancer*, **25**, 21.

King, O. H. & Coleman, S. A. (1965) Analysis of Oral Exfoliative Cytologic Accuracy by Control Biopsy Technique. *Acta cytol.*, **9**, 351.

Mehta, F. S., Daftary, D. K. & Sahiar, B. E. (1970) A Correlative Histocytological Study of Epithelial Atypia in Leukoplakic and Submucous Fibrosis Lesions amongst Indian Villagers in a Mass Screening Programme. *Ind. J. Cancer*, **7**, 18.

Mehta, F. S., Pindborg, J. J. Gupta, P. C. & Daftary, D. K. (1969) Epidemiologic and Histologic Study of Oral Cancer and Leukoplakia among 50,915 Villagers in India. *Cancer, N.Y.*, **24**, 832.

PAPANICOLAOU, G. N. (1954) *Atlas of Exfoliative Cytology*. Cambridge, Mass.: Harvard University Press.

PASTAKIA, H. J. (1955) The Vaginal Smear. *Ind. J. med. Sci.*, **9**, 579.

PINDBORG, J. J., MEHTA, F. S., GUPTA, P. C., DAFTARY, D. K. & SMITH, C. J. (1971) Reverse Smoking in Andhra Pradesh, India: a Study of Palatal Lesions among 10,169 Villagers. *Br. J. Cancer*, **25**, 10.

SHKLAR, G., MEYER, I., CATALDO, E. & TAYLOR, R. H. (1968) Correlated Study of Oral Cytology and Histopathology. Report on 2052 Oral Lesions. *Oral Surg.*, **25**, 61.

UMIKER, W. O., LAMPE, I., RAPP, R. & HINIKER, J. J. (1960) Oral Smears in the Diagnosis of Carcinoma and Pre-malignant Lesions. *Oral. Surg.*, **13**, 897.